# O-53 - QUALITY IMPROVEMENT THROUGH SYSTEM LEADERSHIP: A TUBERCULOSIS REGIONAL STRATEGIC OVERSIGHT GROUP (TB RSOG) TO ENSURE OVERSIGHT, ASSURANCE AND ACCOUNTABILITY ACROSS THE MIDLANDS IN ENGLAND

**DOI:** 10.1093/pubmed/fdag046.047

**Published:** 2026-07-09

**Authors:** Dr Helen Webster, Katie Spence, David Pearce, Kate Duffield, William Proto, Deborah Watson, Professor Mike Wade

**Affiliations:** Department Of Health And Social Care; Department Of Health And Social Care; Department Of Health And Social Care; Department Of Health And Social Care; Department Of Health And Social Care; Department Of Health And Social Care; Department Of Health And Social Care

## Abstract

**Background:**

TB remains a significant public health challenge. In 2024, the West and East Midlands had the 2nd and 4th highest regional rates of TB notification, respectively, including Leicester, the local authority with the highest 3-year average TB notification rate in England. Ongoing NHS transformation risks loss of focus for TB control. TB Control Boards are strategic partnership groups but lack formal accountability.

**Objectives:**

To establish a TB Regional Strategic Oversight Group (TB RSOG) to provide assurance and accountability in the Midlands.

**Methods:**

In 2025, we established a 12-month TB RSOG, chaired by our Regional Director of Public Health, to provide oversight and assurance that systems are working effectively to reduce TB incidence. Membership includes ICB Cluster representatives, Directors of Public Health, UKHSA, regional Public Health and NHSE colleagues. Priority areas were defined by 3-year average TB incidence ≥10/100,000 population (total 10 UTLAs). A situation report (Sit Rep) captures TB epidemiology, GIRFT progress, latent TB infection testing, timely access to diagnosis and treatment, BCG coverage, inclusion health, local priorities, and governance. The TB RSOG reports to the NHSE Regional Executive Team.

**Results:**

Sit Reps have created a regional assurance baseline and enabled structured challenge and escalation. Sustained system focus on TB has highlighted cross-system risks, including diagnostic access, capacity constraints, and pathway variation. To address specific risks, the RSOG has moved to ICB Cluster-themed meetings for areas requiring in-depth scrutiny and support. The Sit Rep approach keeps health inequalities prioritised.

**Conclusions:**

Amidst organisational transformation, TB control requires a system level response; the broad partnership of organisations with statutory and strategic responsibilities for population health. The RDPH role in health protection system governance in this context has been critical and to our knowledge, we are the only region using this RSOG model. The approach is transferable to other regions and priorities.

